# Early Colonoscopy Confers Survival Benefits on Colon Cancer Patients with Pre-Existing Iron Deficiency Anemia: A Nationwide Population-Based Study

**DOI:** 10.1371/journal.pone.0086714

**Published:** 2014-01-22

**Authors:** Chieh-Lin Jerry Teng, Jui-Ting Yu, Yi-Huei Chen, Ching-Heng Lin, Wen-Li Hwang

**Affiliations:** 1 Division of Hematology/Medical Oncology, Department of Medicine, Taichung Veterans General Hospital, Taichung, Taiwan; 2 Department of Medicine, Chung Shan Medical University, Taichung, Taiwan; 3 Department of Life Science, Tunghai University, Taichung, Taiwan; 4 Department of Medical Research, Taichung Veterans General Hospital, Taichung, Taiwan; University of Pisa, Italy

## Abstract

This study aimed to examine the prognostic significance of pre-existing iron deficiency anemia (IDA) and the benefits of early colonoscopy in patients with colon cancer, since these have not been clearly established to date. Using the Taiwanese National Health Insurance Research Database, we retrieved and retrospectively reviewed the records of patients aged ≥55 years who were diagnosed with colon cancer between 2000 and 2005. The patient cohort was divided into two groups: patients with (n = 1,260) or without (n = 15,912) an IDA diagnosis during ≤18 months preceding the date of colon cancer diagnosis. We found that diabetes (27.9% vs. 20.3%, p<0.0001), cardiovascular disease (61.6% vs. 54.7%, p<0.001), and chronic kidney disease (4.6% vs. 2.2%, p<0.0001) were more common among patients with IDA than among those without IDA. The median overall survival times for patients with IDA and those without IDA were 4.6 and 5.7 years, respectively (p = 0.002). Patients who underwent colonoscopy ≤30 days, 31–90, and ≥91 days after IDA diagnosis showed median overall survival times of 5.79, 4.43, and 4.04 years, respectively (p = 0.003). Delayed colonoscopy was an independent factor associated with poor overall survival (adjusted hazard ratio, 1.28; 95% confidence interval, 1.07–1.53; p = 0.01). In conclusion, colon cancer patients with IDA were more likely to experience comorbidities than were those without IDA. Pre-existing IDA was a poor prognostic factor in adult men and postmenopausal women who had colon cancer. Early colonoscopy could improve overall survival possibly by facilitating early diagnosis and treatment.

## Introduction

Colon cancer is one of the most common cancers worldwide. More than 140,000 new colon cancer cases are diagnosed annually in the United States [1]. Adjuvant treatment with a combination of oxaliplatin, fluorouracil, and leucovorin has been shown to improve the disease-free survival and overall survival (OS) rates of patients with stage III colon cancer [2]. For metastatic colon cancer, adding bevacizumab [3] and cetuximab [4] to conventional chemotherapeutic regimens has been shown to confer survival benefits. However, early diagnosis and complete resection remain the cornerstones of colon cancer treatment. Effective screening programs are especially important for early diagnosis. Screening via biennial fecal occult blood tests and flexible sigmoidoscopy reportedly reduce the incidence and mortality of colon cancer [5], [6]. However, localized colon cancer is diagnosed in only 40% of patients, even in countries with high-income populations [7].

Approximately 20% of patients with colon cancer experience gastrointestinal bleeding, likely causing iron deficiency anemia (IDA) [8]. Although IDA is strongly associated with colon cancer in adult male and postmenopausal female patients, not all patients undergo complete investigation upon IDA diagnosis. Several reasons preclude investigation for colon cancer in every adult male or postmenopausal female patient with IDA. One of the reasons is bleeding caused by colon cancer can be intermittent and likely overlooked by patients and physicians. Another precluding consideration is the risk of colonoscopy. In a population-based study, Hamdani et al. reported a 0.06% incidence of colonoscopy-induced colonic perforation [9]. Patients are more susceptible to this complication if they are old, female, or have abdominal pain.

It is unclear whether IDA affects colon cancer staging or patients’ survival outcomes. In the same vein, it is important to investigate whether early colonoscopy likely improves the survival of colon cancer patients with pre-existing IDA. Therefore, we conducted a retrospective, observational study to compare the clinical characteristics and OS of colon cancer patients with pre-existing IDA and those without pre-existing IDA. Staging at diagnosis and overall survival were also assessed in relation to the interval between IDA presentation and definitive malignancy diagnoses.

## Materials and Methods

### Study Population

Our study used patients’ records in the Longitudinal Health Insurance Database, which is based on the Taiwanese National Health Insurance Research Database. The database covers 23 million patients registered between March 1995 and December 2010. More than 99% of the entire population of Taiwan is included in this database, which has comprehensive records of clinical visits for each insured person. Specifically, patients’ data include anonymized identification numbers, demographic characteristics, inpatient and outpatient dates, diagnostic codes (International Classification of Disease, Revision 9, Clinical Modification [ICD-9-CM]), and prescriptions. Details of this population-based database have been described further previously [10]. The institutional review board of Taichung Veterans General Hospital approved this study. The written consent from patients was waived by the approving institutional review board in this study.

### Study Subjects

Study subjects were selected from among patients aged ≥55 years, who were diagnosed with colon cancer (ICD-9-CM code 153) between 2000 and 2005. Patients with carcinoma in situ, previous diagnoses of any malignancies, no catastrophic illness registration, and patients who had no intention-to treat were excluded. Finally, 17,172 patients were included in this study. From this cohort, we identified 1,260 patients in whom IDA (ICD-9-CM codes 280.0, 280.8, and 280.9) was diagnosed within 18 months before the diagnosis of colon cancer. To improve the external validity of our findings, we compared the incidence of IDA among patients with colon cancer to that among individuals without colon cancer who had comparable demographics. The control group comprised patients with no history of cancer. These patients were randomly matched (1∶1) to colon cancer patients from the registry of beneficiaries. Matching was performed according to sex, age (10-year increments), and index year. Details on patient demographics and IDA incidence are presented in Table 1.

**Table 1:** Comparisons of IDA Incidence Among Patients With and Without Colon Cancer.

Gender	Age,years	Without colon cancer		With colon cancer		p-value
		PatientNo.	IDA (%)	PatientNo.	IDA (%)	
**Female**	**55–64**	1882	5 (0.3)	1882	124 (6.6)	<0.0001
	**65–74**	2829	15 (0.5)	2829	257 (9.1)	<0.0001
	**≥75**	2699	22 (0.8)	2712	304 (11.2)	<0.0001
	**Total**	7410	42 (0.6)	7423	685 (9.2)	<0.0001
**Male**	**55–64**	2442	2 (0.1)	2442	111 (4.6)	<0.0001
	**65–74**	3904	18 (0.5)	3904	225 (5.8)	<0.0001
	**≥75**	3341	15 (0.5)	3403	239 (7.0)	<0.0001
	**Total**	9687	35 (0.4)	9749	575 (5.9)	<0.0001
**Total**		17097	77 (0.5)	17172	1260 (7.3)	<0.0001

IDA: iron deficiency anemia; No.: number.

### Comorbidities and Outcome Measures

Major comorbidities at baseline were treated as covariates and included hypertension (ICD-9-CM codes 401–405), diabetes (ICD-9-CM code 250), hyperlipidemia (ICD-9-CM code 272), cardiovascular disease (ICD-9-CM codes 390–438), and chronic kidney disease (ICD-9-CM code 585). The index date for each subject was defined as the date of the first colon cancer diagnosis. The study endpoints were date of death, date of exclusion from the database, or the study cut-off date (end of 2010). Stage I/II disease was considered in patients who had neither lymph node nor distant metastases. Patients who had either lymph node or distant metastases were deemed to have stage III/IV disease. To further validate the staging criteria, we compared stage distribution in current study with Taiwan’s national cancer registration data (Table S1), and found that this was comparable.

### Statistical Analysis

The chi-square test and analysis of variance were used, as appropriate. Multivariate Cox proportional-hazards regression was used to estimate the effect of the interval between IDA diagnosis and colonoscopic examination on the risk of mortality, in terms of hazard ratios and accompanying 95% confidence intervals (CIs). Differences in median survival times were analyzed using the log-rank test. Two-tailed *p*-values of <0.05 were considered statistically significant. All statistical analyses were performed using SAS statistical software (version 9.2 for Windows; SAS Institute, Inc., Cary, NC, USA).

## Results

### Clinical Characteristics of Colon Cancer Patients with IDA and Those without IDA

Patients with colon cancer were grouped into those with (n = 1,260) or without IDA (n = 15,912) diagnosed during the 18 months preceding the colon cancer diagnosis (Table 2). Colon cancer patients with pre-existing IDA and those without pre-existing IDA were aged 72.7±8.5 and 70.8±8.5 years on average, respectively. Patients with IDA were significantly older than were those without IDA (*p*<0.0001). Moreover, colon cancer patients with IDA were more likely to be female (54.4% vs. 42.4%, *p*<0.0001) (Table 2) than were those without IDA. Patients with IDA were also more likely to have comorbidities, including diabetes (27.9% vs. 20.3%, *p*<0.0001), cardiovascular disease (61.6% vs. 54.7%, *p*<0.001), and chronic kidney disease (4.6% vs. 2.2%, *p*<0.0001), than those without IDA. However, the prevalence of stage III/IV colon cancer was not significantly different between patients with IDA and those without IDA (71.3% vs. 70.5%, respectively; *p* = 0.54).

**Table 2:** Clinical Characteristics of Colon Cancer Patients With and Without Pre-existing IDA.

Variable	Without IDA	With IDA	Total	p-value
	(n = 15912)	(n = 1260)	(n = 17172)	
	Number (%)	Number (%)	Number (%)	
**Age, years (mean ± SD)**	70.8±8.5	72.7±8.5	70.9±8.5	<0.0001^a^
55–64	4089 (25.7)	235 (18.7)	4324 (25.2)	
65–74	6251 (39.3)	482 (38.2)	6733 (39.2)	
**≥**75	5572 (35.0)	543 (43.1)	6115 (35.6)	
**Gender**				<0.0001^b^
Female	6738 (42.4)	685 (54.4)	7423 (43.2)	
Male	9174 (57.6)	575 (45.6)	9749 (56.8)	
**Hypertension**	7193 (45.2)	604 (47.9)	7797 (45.4)	0.06^b^
**DM**	3236 (20.3)	351 (27.9)	3587 (20.9)	<0.0001^b^
**Hyperlipidemia**	2325 (14.6)	182 (14.4)	2507 (14.6)	0.87^b^
**CVD**	8704 (54.7)	776 (61.6)	9480 (55.2)	<0.0001^b^
**CKD**	352 (2.2)	58 (4.6)	410 (2.4)	<0.0001^b^
**Stage**				0.54^b^
I/II	4701 (29.5)	362 (28.7)	5063 (29.5)	
III/IV	11211 (70.5)	898 (71.3)	12109 (70.5)	

IDA: iron deficiency anemia; SD: standard deviation; DM: diabetes mellitus; CVD: cardiovascular diseases; CKD: chronic kidney disease.

a indicates an ANOVA test;

b indicates a chi-square test.

### Colon Cancer Patients with Pre-existing IDA Had Inferior OS

**Figure 1:**
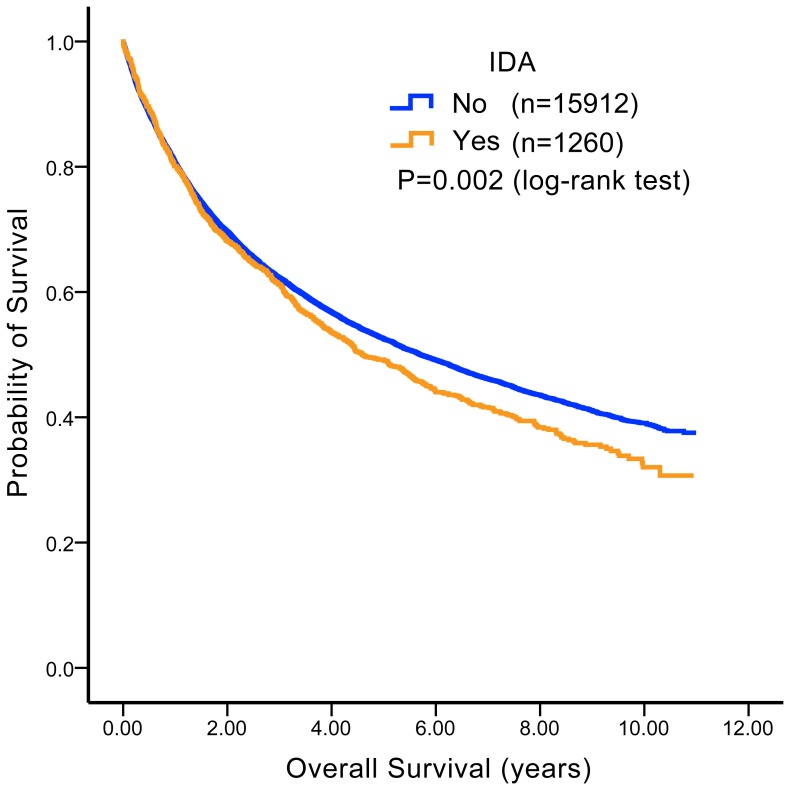
Overall survival (OS) comparison among colon cancer patients with and without iron deficiency anemia (IDA). Among adult male and postmenopausal female colon cancer patients, median OS times were 4.6 and 5.7 years for patients with per-existing IDA and those without per-existing IDA, respectively. Patients with pre-existing IDA had inferior OS compared to those without pre-existing IDA (*p* = 0.002).

To investigate the value of IDA as a biomarker for predicting patient outcomes, we compared the OS of colon cancer patients with pre-existing IDA and those without pre-existing IDA. The median OS times were 4.6 and 5.7 years for patients with IDA and those without IDA, respectively. As presented in Figure 1, the OS of patients with pre-existing IDA was worse than the OS of patients without IDA (*p* = 0.002).

### Early Colonoscopy Did Not Result in an Increased Number of Stage I/II Diagnoses

To investigate whether early colonoscopy in colon cancer patients with pre-existing IDA could lead to cancer diagnosis at early disease stages, we divided our study cohort into 3 groups according to the interval between IDA diagnosis and colon cancer confirmation. Among the 1,015 patients, 489 (48.2%), 143 (14.1%), and 383 (37.7%) underwent colonoscopy at ≤30, 31–90, and ≥91 days after IDA diagnosis (Table 3). The median interval between IDA diagnosis and colonoscopy was 36 days (range: 0–533 days). Patients who underwent early colonoscopy were slightly younger (*p = *0.0025) than other patient groups. Several comorbidities, including hypertension (*p* = 0.0001), cardiovascular disease (*p*<0.001), and chronic kidney disease (*p* = 0.04), were found more commonly among patients who underwent colonoscopy ≥91 days after IDA diagnosis. However, there was no statistically significant difference in the prevalence of diabetes (*p* = 0.06) or hyperlipidemia (*p* = 0.07) between the two groups.

**Table 3:** Clinical Characteristics According to Interval Between IDA and Colon Cancer Diagnosis.

	Days of colonoscopic examinations after IDA diagnosis			p-value
	0–30	31–90	≥91	
	(n = 489)	(n = 143)	(n = 383)	
	Number (%)	Number (%)	Number (%)	
**Age, years (mean ± SD)**	71.9±8.8	73.0±7.8	73.9±8.1	0.003^a^
55–64	109 (22.3)	25 (17.5)	47 (12.3)	
65–74	176 (36.0)	56 (39.2)	157 (41.0)	
**≥**75	204 (41.7)	62 (43.4)	179 (46.7)	
**Gender**				0.97^b^
Female	264 (54.0)	78 (54.5)	210 (54.8)	
Male	225 (46.0)	65 (45.5)	173 (45.2)	
**Hypertension**	213 (43.6)	64 (44.8)	220 (57.4)	0.0001^b^
**DM**	122 (24.9)	37 (25.9)	123 (32.1)	0.06^b^
**Hyperlipidemia**	62 (12.7)	23 (16.1)	70 (18.3)	0.07^b^
**CVD**	262 (53.6)	88 (61.5)	281 (73.4)	<0.0001^b^
**CKD**	14 (2.9)	5 (3.5)	24 (6.3)	0.04^b^
**Stage**				0.95^b^
I/II	140 (28.6)	39 (27.3)	108 (28.2)	
III/IV	349 (71.4)	104 (72.7)	275 (71.8)	

IDA: iron deficiency anemia; SD: standard deviation; DM: diabetes mellitus; CVD: cardiovascular disease; CKD: chronic kidney disease.

a indicates an ANOVA test;

b indicates a chi-squared.

In addition, stage I/II disease was found in 28.6%, 27.3%, and 28.2% of patients who underwent colonoscopy ≤30, 31–90, and ≥91 days after IDA diagnosis, respectively (Table 3; *p* = 0.95). Stage III/IV colon cancer was diagnosed in >70% of patients, regardless of colonoscopy timing.

### Early Colonoscopy Improved OS

**Figure 2:**
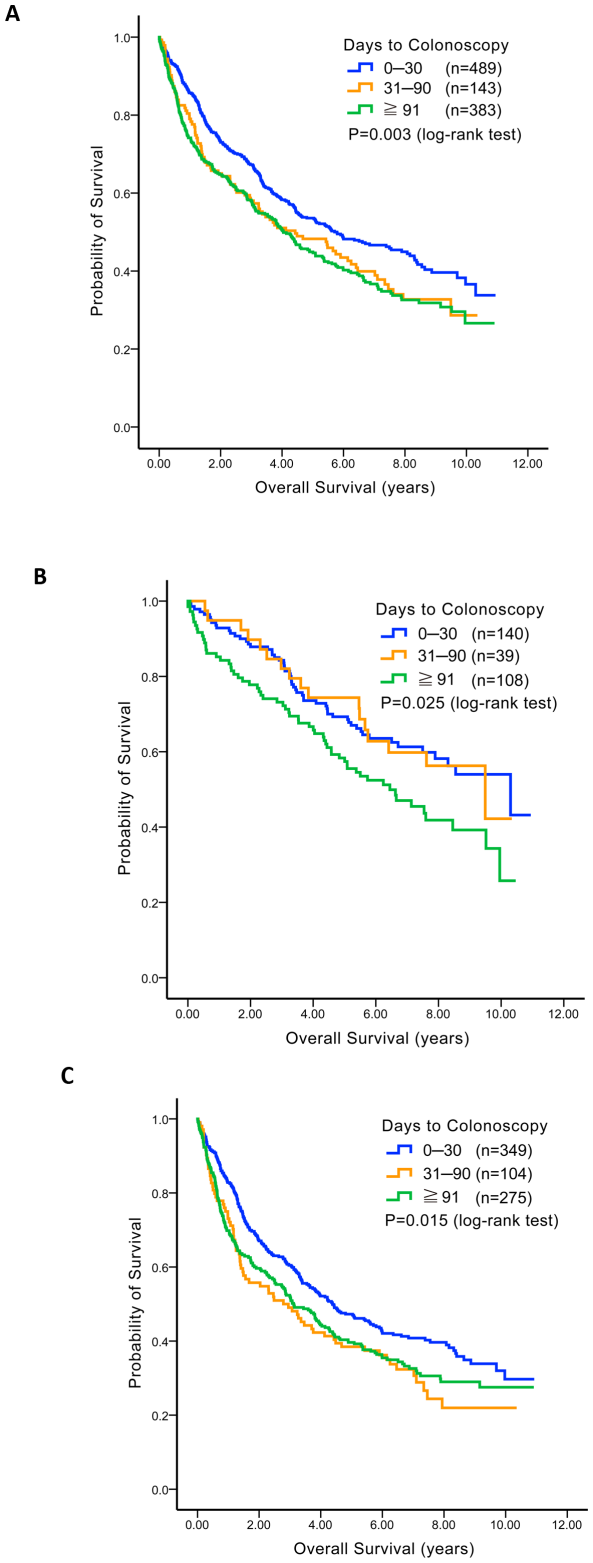
Survival comparison according to interval between diagnosis of iron deficiency anemia (IDA) and colon cancer. For colon cancer patients with pre-existing IDA, their overall survival times were compared according to the interval between IDA diagnosis and colon cancer confirmation (≤30 days, 31–90 days, and ≥91 days) and are presented with Kaplan–Meir survival curves. (A) For all patients together, (B) for those with stage I/II disease, and (C) for those with stage III/IV disease.

We investigated whether early colonoscopy could confer a survival benefit in the same three groups of patients with colon cancer and pre-existing IDA. Our results are presented in Figure 2A. The median OS times were 5.79, 4.43, and 4.04, respectively, when colonoscopy was performed ≤30, 31–90, and ≥91 days after IDA diagnosis (*p* = 0.003).

Patient subgroups were analyzed to investigate which subgroup benefited the most from early colonoscopy in terms of survival (Figure 2B). Median OS times were 10.30, 9.49, and 6.45 years (*p* = 0.025) among patients with stage I/II colon cancer (n = 287) when colonoscopy was performed ≤30 days (n = 140), 31–90 days (n = 39), and ≥91 days (n = 108) before IDA diagnosis, respectively (Figure 2B). For patients with stage III/IV colon cancer (n = 738), the median OS times were 4.38 (n = 349), 2.79 (n = 104), and 3.12 (n = 275) years (*p* = 0.015) in these three subgroups (Figure 2C).

### Delayed Colonoscopy was Independently Associated with Unfavorable OS

The unadjusted analysis presented above could not clarify whether the observed survival benefits resulted from early colonoscopy or potential confounders, including patient age and comorbidities. Therefore, we performed a Cox-regression analysis after adjusting for age, gender, hypertension, cardiovascular disease, chronic kidney disease, diabetes, and hyperlipidemia (Table 4). For all patients (irrespective of disease stage), colonoscopy performed ≥91 days after IDA diagnosis had a hazard ratio of 1.28 (95% CI: 1.07–1.53, *p* = 0.01). For patients who had stage I/II colon cancer, the hazard ratio for performing colonoscopy ≥91 days after IDA diagnosis was 1.52 (95% CI: 1.05–2.20, *p* = 0.03); for stage III/IV colon cancer, this was 1.22 (95% CI: 1.00–1.49, *p* = 0.05).

**Table 4:** Hazard Ratios For Death According to Interval Between IDA and Colon Cancer Diagnosis, Adjusted For Covariates* Using A Cox Proportional Hazards Model.

Variable		Hazard ratio	95% CI		p-value
			Lower	Upper	
**Stage I/II (n = 287)**					
Interval from IDA to colon cancer diagnosis (days)	0–30	1.00	–	–	
	31–90	1.16	0.67	2.01	0.60
	≥91	1.52	1.05	2.20	0.03
**Stage III/IV (n = 728)**					
Interval from IDA to colon cancer diagnosis (days)	0–30	1.00	–	–	
	31–90	1.35	1.03	1.76	0.03
	≥91	1.22	1.00	1.49	0.05
**All stages (n = 1015)**					
Interval from IDA to colon cancer diagnosis (days)	0–30	1.00	–	–	
	31–90	1.26	0.99	1.59	0.06
	≥91	1.28	1.07	1.53	0.01

IDA: iron deficiency anemia; CI: confidence interval.

* Age, gender, hypertension, diabetes mellitus, hyperlipidemia, cardiovascular disease, and chronic kidney disease.

## Discussion

Iron deficiency anemia is uncommon in adult men and postmenopausal women. In the United States, IDA prevalence is only 2–5% in the abovementioned group [11]. In our study cohort, IDA was diagnosed in <1% of individuals aged >55 years who did not develop colon cancer. In contrast, nearly 10% of patients with colon cancer developed IDA within 18 months preceding cancer diagnoses. Our results also indicated that diabetes, cardiovascular disease, and chronic kidney disease are more common among colon cancer patients with IDA than among those without IDA. Previous epidemiological studies have reported essentially similar results, indicating that anemic patients have a high risk of cardiovascular disease [12], chronic kidney failure, diabetes [13], acute coronary syndrome [14], and heart failure [12]. Hsu et al. [15] further demonstrated that IDA patients older than 65 years were at an increased risk of all-cause mortality. These comorbidities may partially explain why IDA could predict poor survival in our cohort of colon cancer patients. However, the pathophysiological mechanisms underlying the association between IDA and a high incidence of comorbidities in the colon cancer patients are not fully understood.

In addition to excessive blood loss, deregulated iron metabolism could partly explain this relationship. A cross-sectional survey by Thomas et al. [16] indicated that, among patients with diabetes and chronic kidney disease, failure of kidneys to produce erythropoietin in response to reduced hemoglobin levels is a key factor causing anemia. Moreover, autonomic neuropathy, nephritic syndrome, reduced red blood cell half-life, and medication potentially contribute to anemia in patients with any of these disorders [17], [18]. These observations indirectly explain and support our conclusion that the incidences of diabetes, cardiovascular disease, and chronic kidney disease were higher among colon cancer patients with pre-existing IDA than among those without IDA.

Retrospective [19] and prospective [20] studies have shown that rectal bleeding as an initial presentation of colon cancer is associated with an early disease stage and better survival. However, our study did not support the hypothesis that IDA would be a useful biomarker for early colon cancer diagnosis. In contrast, <30% of patients with IDA had stage I/II colon cancer, irrespective of colonoscopy timing. This result indicated that, as compared with the stool occult blood testing, hemograms and iron profiling may be more specific methods for colon cancer screening in adult men and postmenopausal women (specificity: 99.5%). However, based on the evidence presented by our study, such testing may not be sufficiently sensitive (sensitivity: 7.3%). Indeed, colon cancer stage distribution did not vary according to the timing of colonoscopy.

Interestingly, earlier colonoscopy was significantly associated with improved OS rates in our study cohort, both for stage I/II and III/IV colon cancer patients. Because the patients who underwent an earlier colonoscopy were younger and had fewer comorbidities than those who underwent a later colonoscopy, their improved medical conditions were likely partially responsible for this outcome. However, after adjusting for age, gender, hypertension, diabetes, hyperlipidemia, cardiovascular disease, and chronic kidney disease, early colonoscopy emerged as an independent prognosticator for favorable survival in colon cancer patients with pre-existing IDA (Table 4). One possibility is that earlier colonoscopy could decrease the likelihood of micrometastases. Lymph node micrometastases are reported to be associated with high chances of colon cancer recurrence and poor disease-free survival [21], [22]. In our cohort, early colonoscopy could have resulted in the diagnosis of more actual stage I and II cases, which were misdiagnosed because of micrometastases to adjacent lymph nodes. However, further studies are necessary to support this hypothesis. Additionally, timely surgical interventions and/or chemotherapy (either adjuvant or palliative) are other possibilities. Better management of colon cancer by hospitals that facilitate access to earlier colonoscopy needs to be taken into consideration as well.

Imprecise recording of disease stage was one of the major limitations of this study. Comparable stage distributions between Taiwan’s national cancer registration data and data on our study cohort could be a validating factor, although this agreement was not perfect. Additionally, lead-time bias needs to be taken into consideration in this study as well. Observational studies with larger cohorts and randomized controlled studies are needed to resolve this clinical dilemma.

In conclusion, our study demonstrated that pre-existing IDA was an independent factor associated with poor survival outcomes in adult men and postmenopausal women with colon cancer. Inferior survival outcomes could be due to a higher prevalence of diabetes, cardiovascular disease, and chronic kidney disease. Although timely colonoscopy did not lead to a diagnosis of colon cancer at early stages (stage I/II), it improved the OS of patients with both stage I/II and III/IV disease possibly by facilitating early diagnosis and treatment. When IDA is diagnosed in adult men and postmenopausal women, both patients and clinicians should carefully consider the possibility of undiagnosed colon cancer.

## Supporting Information

Table S1 **Stage distribution in colon cancer patients** ≥**50 years from Taiwan’s national cancer registration data.** To further validate the staging criteria in this study, we compared stage distribution in our study cohort with that from Taiwan’s national cancer registration data, and found that this was comparable, which suggested the staging criteria in current study was reliable. No.: number.
(DOC)
