# Supplementary material for: Early Colonoscopy Confers Survival Benefits on Colon Cancer Patients with Pre-Existing Iron Deficiency Anemia: A Nationwide Population-Based Study
Source: PLoS One. 2014 Jan 22;9(1):e86714. doi: 10.1371/journal.pone.0086714 (PMC3899285; doi:10.1371/journal.pone.0086714)
Supplement: Table S1 — Stage distribution in colon cancer patients ≥50 years from Taiwan’s national cancer registration data. To further validate the staging criteria in this study, we compared stage distribution in our study cohort with that from Taiwan’s national cancer registration data, and found that this was comparable, which suggested the staging criteria in current study was reliable. No.: number. (DOC) [file pone.0086714.s001.doc]

| **Table S1. Stage Distribution in Colon Cancer Patients** ≥ **50 Years from Taiwan’s National Cancer Registration Data** | | | | | |
| --- | --- | --- | --- | --- | --- |
|  | **2006**  **No. (%)** | **2007**  **No. (%)** | **2008**  **No. (%)** | **2009**  **No. (%)** | **2010**  **No. (%)** |
| **Stage I** | 491 (13.9) | 583 (14.0) | 560 (13.0) | 724 (14.4) | 1047 (17.4) |
| **Stage II** | 1072 (30.3) | 1236 (29.8) | 1267 (29.5) | 1439 (28.6) | 1652 (27.5) |
| **Stage III** | 920 (26.0) | 1116 (26.9) | 1180 (27.5) | 1424 (28.3) | 1692 (28.2) |
| **Stage IV** | 840 (23.7) | 1032 (24.9) | 1108 (25.8) | 1254 (24.9) | 1398 (23.3) |
| **Unknown** | 218 (6.2) | 184 (4.4) | 182 (4.2) | 197 (3.9) | 221 (3.7) |
| **Total** | 3541 (100) | 4151 (100) | 4297 (100) | 5038 (100) | 6010 (100) |
| No.: number | | | | | |
